# Knowledge and attitudes towards antibiotic use and resistance among undergraduate healthcare students at University of Rwanda

**DOI:** 10.1186/s40545-020-00207-5

**Published:** 2020-04-22

**Authors:** Lyduine Nisabwe, Hirwa Brice, Marie Christine Umuhire, Olivia Gwira, Jean De Dieu Harelimana, Zephanie Nzeyimana, Osee Rurambya Sebatunzi, Emmanuel Kamanzi Rusingiza, Innocent Hahirwa, Claude Mambo Muvunyi

**Affiliations:** 1 Department of Pharmacy, School of Medicine and Pharmacy, College of Medicine and Health Sciences, University of Rwanda, Kigali, Rwanda; 2 Department of Biomedical Laboratory Science, School of Health Science, College of Medicine and Health Sciences, University of Rwanda, Kigali, Rwanda; 3 Department of Medical Laboratory Sciences, Mount Kenya University, Kigali, Rwanda; 4 Department of Internal Medicine, School of Medicine and Pharmacy, College of Medicine and health Sciences, University of Rwanda, Kigali, Rwanda; 5 Department of Pediatrics, School of Medicine and Pharmacy, College of Medicine and health Sciences, University of Rwanda, Kigali, Rwanda; 6 Department of Clinical Biology, School of Medicine and Pharmacy, College of Medicine and Health Sciences, University of Rwanda, Kigali, Rwanda

**Keywords:** Antibiotic use, Antimicrobial resistance, Undergraduate students, Knowledge, And attitude

## Abstract

**Background:**

Antimicrobial resistance (AMR) is an imminent threat to modern medicine. As the efficacy of treatment regimens is reduced, mortality and morbidity attributed to infectious diseases is expected to rise dramatically across the globe. Antimicrobial stewardship and good prescription practices are critical to conserving available therapeutics; it is appropriate, therefore, to appraise our attitudes and knowledge of antimicrobial resistance, particularly for the future healthcare practitioners.

**Methods:**

This is a descriptive cross-sectional study that was conducted among 282 medicals, dental and pharmacy students from the University of Rwanda. Questionnaires were used to collect data from the 4th to 29th March 2017.

**Results:**

Students from Level 3 to level 6 have demonstrated a good knowledge on antibiotics and antimicrobial resistance. Generally, 95% (*n* = 218) agreed that the inappropriate use of antibiotics could lead to antibiotic resistance. It was found that 96% (*n* = 220) of the respondents had heard about AMR outside their degree courses. 49% (*n* = 112) of the participants reported that they are able to purchase antibiotics without a prescription. 96% (n = 220) agreed that it was important for healthcare students to be knowledgeable about antimicrobial resistance. Perhaps most surprisingly, it was found that 83% (*n* = 191) of participants were unfamiliar with the concept of antimicrobial stewardship and 49% (*n* = 21) had not yet discussed antimicrobial resistance as part of their education, albeit only 1% (*n* = 3) was completely unfamiliar with the term. Furthermore, 38% (*n* = 86) did not support that the antibiotics were overused in Rwanda, 23% (*n* = 10) did not agree that inappropriate antimicrobial use contributed to antimicrobial resistance, and 50% (*n* = 22) of participants agreed that antibiotics were indicated in the treatment of pain and inflammation.

**Conclusions:**

The present study reports a moderate knowledge on AMR among the healthcare students. The gaps in the current formal training of healthcare individuals have been identified as well. We highlight the necessity to enhance educational approaches to introduce the key concepts of AMR and antimicrobial stewardship to the curriculum of healthcare students.

## Introduction

Antimicrobial resistance is a natural biological phenomenon when microbes are no longer killed by an antimicrobial to which they were previously sensitive, often due to the genetic mutations of microorganisms [1, 2]. This phenomenon frequently occurs when the antimicrobials are misused. The resistant mutants are often considered as “superbugs” [3].

Antimicrobial resistance is a global crisis that requires global coordinated efforts. Although the *WHO’s Global action plan on antimicrobial resistance is being formulated* [4]*.* The current studies have reported dramatic increase of antimicrobial resistance especially in resources limited countries [5]. There is a growing body of literature that accents the significance of resistance to antibiotics that are commonly prescribed in lagging countries [6]. Therefore, there is an urgent need to address antimicrobial resistance in developing countries especially in Africa due to the fact that it is being overlooked and yet highly linked high morbidity and mortality [7]. A great demand of broad-spectrum agents due to ineffectiveness of certain antibiotics on which specific pathogens have developed resistance [6, 8], poses an economic burden on developing countries [7].

A study conducted among medical students in Democratic republic of Congo (DRC) showed that medical students are aware of antibiotics overuse and that it would be of significance to raise awareness of antibiotics among these future prescribers [9]. *Evidence suggests that strategic actions such as antimicrobial stewardship must be enforced.* Another study carried out in India has revealed a gap in the knowledge regarding antibiotic use among medical and dental students. Thus, they recommended to reinforce the curriculum by introducing more lectures on antimicrobial stewardship [10].

Furthermore, A study conducted at a tertiary healthcare hospital in Rwanda, reported high rate of AMR and highlighted the need for rational use of antibiotics, implementation of antimicrobial surveillance programs, and appropriate infection control measures to tackle AMR [11].

In some countries such as Albania and Pakistan, self-purchasing of antibiotic was predominant among fresh pharmacists [12, 13]. A study conducted among undergraduate and postgraduate pharmacy students in southern India highlighted the need to integrate specific lectures on appropriate use of antibiotics in pharmacy programs [14]. Similarly, findings from other studies conducted in South Africa and Bangladesh, stated that training opportunities and standardized curricula on antimicrobial stewardship, as educational interventions, would boost the knowledge of medical students and strengthen their confidence on antimicrobial prescribing [15, 16].

Different studies have associated antimicrobial resistance with the level of knowledge, attitudes and perceptions on antimicrobial use in healthcare provider while others have highlighted patient pressure and failure to complete the full course of treatment as two main factors that contribute to drug resistance [5, 17]. Healthcare students as future prescribers and dispensers of antimicrobials may have a different perspective on the matter. Globally, self-medication remains at the top of the main cause of AMR because antibiotics are still being over-the-counter dispensed in many settings [18, 19].

Limited evidence from African countries suggests that self-medication with antibiotics is a common health-seeking behavior and patients directly reach out to pharmacists without medical consultation due to the inability of affording healthcare services [20] . The challenge of having a limited number of certified pharmacists able to provide proper patient counselling on the use of antimicrobials is another raison for irrational use of antibiotics [21].

Therefore, improving the knowledge of healthcare students especially pharmacy students is crucial in the fight against AMR [22]. In addition, pharmacists are well placed to forefend AMR by encouraging the rational use of antimicrobials as reported by WHO [23]. Well educated pharmacists can contribute to the reduction of irrational use of antimicrobials, hence improving pharmacist’s role in the healthcare system of developing countries is decisive in the battle against AMR globally [24].

It is important to highlight that in Rwanda, healthcare students are allowed to practice right after their graduation. Thus, it would be of significance to know their knowledge regarding antimicrobials use. The present study aimed to evaluate the level of knowledge, attitudes and perceptions of healthcare students towards antimicrobial use and resistance in Rwanda.

## Materials and methods

### Study setting, design and population

This was a descriptive cross-sectional study that included Medicine, pharmacy and dentistry students from the University of Rwanda. Sample size was determined by using the formula below considering the *P* value of 50% for antibiotics use giving total sample size of 282.

$$ \mathrm{n}=\left[{\mathrm{z}}^2\ast \mathrm{p}\ast \left(1-\mathrm{p}\right)/{\mathrm{e}}^2\right]/\left[1+\left({\mathrm{z}}^2\ast \mathrm{p}\ast \left(1-\mathrm{p}\right)/\left({\mathrm{e}}^2\ast \mathrm{N}\right)\right)\right] $$

Where: *N* = 1051 (population size); z = 1.96 for a confidence level (α) of 95%, *p* = 0.5 proportion (expressed as a decimal); e = margin of error.

$$ \mathrm{z}=1.96,\mathrm{p}=0.5,\mathrm{N}=1051,\mathrm{e}=0.05 $$

$$ \mathrm{n}=\left[{1.96}^2\ast 0.5\ast \left(1-0.5\right)/{0.05}^2\right]/\left[1+\left({1.96}^2\ast 0.5\ast \left(1-0.5\right)/\left({0.05}^2\ast 1051\right)\right)\right] $$

$$ n=384.16/1.3655=281.329 $$

$$ \mathrm{n}\approx 282 $$

Stratified sampling technique was used to select representative classes. The total sample size of 229 participants was distributed among selected classes according to class size. Finally, the study subjects were selected using systematic random sampling technique. 229 participants who responded to the questionnaire.

### Data collection tool

A questionnaire of both close-ended and open-ended questions from other studies were adapted and adjusted and administered to participants to collect relevant information. The questionnaire consisted of four sections. The first section explored the demographic characteristics and information related to antibiotic use. The second section comprised questions aiming to evaluate knowledge of participants about antimicrobials and resistance. The third section was to assess the resources for Antimicrobial resistance awareness. The last part was made of sixteen questions aiming to assess the attitudes and perceptions towards antimicrobial use and prescription. Introduction of the study and its objectives was done prior to distribution of questionnaires to the participants.

### Data analysis

Prior to data analysis, questions were grouped according to assessed parameters: respondent characteristics, knowledge about antibiotics and resistance, source of information on antimicrobial resistance, attitudes and perceptions regarding antimicrobial use and resistance.

Data were captured and analyzed using both Statistical Package for Social Sciences (SPSS V.16.0) software and Microsoft Excel 2010.

### Ethical consideration

Kigali University Teaching Hospital ethical committee approved the research proposal and the permission from the University of Rwanda was also granted. Participation of the present study was voluntary, and each participant signed a consent form.

## Results

Table 1 shows details on demographic characteristics of the study population. Females were 40.17% and males were 59.83%. The half of respondents was medical students while dentistry was the least represented (18%). Respondents were from all class levels with first 2 years representing 44% of participants. As far as the use of antibiotics in the previous year is concerned, 41% of students reported that they had used antibiotics one to two times and 13% admitted having used them three to five times. A relatively small number of respondents (7%) reported to have used antibiotics more than five times in the previous year while 31% of participants never used them. Almost two-thirds of participants had no family member working in Health field.

**Table 1:** Respondents’ distribution

Respondents’ characteristics	Frequency	Percentage
Gender		
Male	137	59.83
Female	92	40.17
Degree course/Department		
Medicine	115	50.22
Dentistry	41	17.9
Pharmacy	73	31.88
Year of study/Level		
First	51	22.27
Second	50	21.83
Third	31	13.54
Fourth	52	22.71
Fifth	30	13.1
Sixth	15	6.55
Do you have a family in health field?		
Yes	86	37.55
No	143	62.45
How often have you used antibiotics?		
1–2 times	93	40.61
3–5 times	29	12.66
More than 5 times	17	7.42
Never	90	39.3

As it can be depicted from Table 2, the highest percentage of fully correct answers (“Strongly agree” and “Strongly disagree”) was around 62%. Only 41.9% of respondents disagreed with this statement “*Antibiotics are indicated to reduce any kind of pain and inflammation*”.

**Table 2:** Knowledge of respondents on Antibiotics

Knowledge statement	Strongly agree (%)	Agree (%)	Uncertain (%)	Disagree (%)	Strongly disagree (%)
Antibiotics are useful for bacterial infections (e.g. Tuberculosis)	143(62.4)	67(29.3)	4(1.7)	8(3.5)	7(3.1)
Antibiotics are useful for viral infections (e.g.flu).	10(4.4)	33(14.4)	15(6.6)	48(21.0)	123(53.7)
Antibiotics are indicated to reduce any kind of pain and inflammation.	11(4.8)	39(17)	22(9.6)	61(26.6)	96(41.9)
Antibiotics can kill good bacteria or normal flora present in our organism	121(52.8)	77(33.6)	9(3.9)	13(5.7)	9(3.9)
Antibiotics can cause allergic reactions	102(44.5)	83(36.2)	22(9.6)	17(7.4)	5(2.2)
Antibiotics can cause secondary infections	103(45)	105(45.9)	8(3.5)	3(1.3)	10(4.4)

As it is described in Table 3, respondents demonstrated a remarkable and satisfactory knowledge in identifying whether Amoxicillin and Penicillin were antibiotics or not, where more than 90% responded correctly to related statement. However, surprisingly 20% of the participants classified Aspirin among antibiotics.

**Table 3:** Knowledge of respondents on Antibiotics- Identification of antibiotics

Knowledge statement	Yes (%)	No (%)	Don’t Know (%)
Penicillin or Amoxicillin are antibiotics	216(94.3)	8(3.5)	5(2.2)
Aspirin is an antibiotic	38(16.6)	184(80.3)	7(3.1)
Paracetamol is an antibiotic	13(5.7)	213(93.0)	3(1.3)

As it is shown in Table 4, the responses obtained when participants were asked whether an inappropriate use of antimicrobials could cause antimicrobial resistance were quite interesting and promising as this was confirmed by 95% of the respondents. The great majority of respondents (89.5%) admitted that poor infection control practices contribute to Antimicrobial resistance while 59.8% agreed that antimicrobials are overused in Rwanda. Surprisingly none of the respondents knew what antimicrobial stewardship is and even the 34 participants who answered yes to the question, failed to correctly explain its meaning.

**Table 4:** Knowledge on antimicrobial resistance

Knowledge question	Yes (%)	No (%)
Are antimicrobials overused in Rwanda?	137(59.8)	86(37.6)
Can inappropriate use of antimicrobials cause antimicrobial resistance?	218(95.2)	11(4.8)
Do you think it is important for the prescriber to do an antibiogram before prescribing antibiotic?	202(88.2)	26(11.4)
Do poor infection control practices contribute to antimicrobial resistance?	205(89.5)	23(10)
Do you know what antimicrobial stewardship means?	34(14.8)	191(83.4)

The following Table 5, details the respondents’ views on whether antibiotics can be used to reduce any kind of pain and inflammation, this statement was used to evaluate students’ knowledge on antibiotics according to their level of study and programs. The majority of the right answers to the related were from higher levels of study (level 3 to 6) indicting with no surprise, that students in lower levels have a low level of knowledge regarding antibiotics. In addition to that, as shown in Table 5, the rate of correct answers was higher in Pharmacy students compared to medical and dental students.

**Table 5:** Results of the question “Antibiotics are indicated to reduce any kind of pain or inflammation” according to the year of studies and department

Department	Level	Strongly disagree (%)	Disagree (%)	Uncertain (%)	Agree (%)	Strongly agree (%)
Medicine	Pre-clinical (*n* = 39)	13(33.3)	12(30.8)	5(12.8)	5(12.8)	4(10.2)
	Clinical (*n* = 50)	31(62)	13(26)	2(4)	3(6)	1(2)
Pharmacy	Low level (*n* = 43)	27(62.8)	11(25.7)	2(4.6)	2(4.6)	1(2.3)
	Advanced level (*n* = 42)	42(100)	0(0)	0(0)	0(0)	0(0)
Dentistry	Low level (*n* = 23)	4(17.4)	5(21.8)	1(4.3)	9(39.1)	4(17.4)
	Advanced level *n* = 32	12(37.5)	12(37.5)	5(15.6)	2(6.25)	1(3.1)

As it is recapitulated in Table 6, almost all respondents reported to have heard about antimicrobial resistance before, 78.9% of said that they have discussed about it during degree courses. Among the respondents 96.1% indicated that they had heard about AMR outside degree courses.

**Table 6:** Sources of information on AMR awareness

Knowledge statement	Yes (%)	No (%)
Have you ever heard about antibiotic resistance?	226(98.7)	3(1.3)
Have you discussed about antibiotic resistance during your course?	180(78.6)	49(21.4)
Do you have a course of antimicrobial resistance in your curriculum?	141(61.4)	88(38.4)
Have you ever heard about AMR out of your degree courses?	220(96.1)	9(3.9)
Do you think healthcare students should be knowledgeable about AMR?	220(96.1)	7(3.1)
Do you think general knowledge should be considered when prescribing antibiotics?	210(91.7)	10(4)

Table 7 describes the attitudes and perceptions regarding antimicrobial use and resistance. Only 15% reported that the usually use antibiotics for fever whereas 27% admitted that they have used antibiotics to treat cold or sore throat. Around three-quarters reported that they don’t keep leftovers antibiotics for a possible future use.

**Table 7:** Respondents’ attitudes and perceptions towards antimicrobials use and resistance

Statement	Yes (%)	No (%)
Do you usually take antibiotic for cold or sore throat	62(27.1)	162(70.7)
Do you take antibiotic for fever?	35(15.3)	193(84.3)
Do you usually stop taking antibiotic when you start feeling better	34(14.8)	195(85.2)
Do you take antibiotic only when prescribed by the doctor?	137(59.8)	91(39.7)
Do you keep leftover antibiotics at home because they might be useful in the future?	46(20.1)	183(79.9)
Do you use leftover antibiotics when you have cold, sore throat or flu without consulting your doctor?	32(14.0)	196(85.6)
Have you shared antibiotics with another member of your family?	48(21.0)	180(78.6)
Can you buy antibiotics without a medical prescription?	112(48.9)	117(51.1)
Have you ever started an antibiotic therapy after a simple doctor call, without a proper medical examination?	153(66.8)	76(33.2)

Self-medication was reported by almost half of the participants who admitted having purchased antibiotics without a medical prescription.

## Discussion

This study aimed to evaluate the knowledge and attitudes towards antibiotic use and resistance among healthcare students in Rwanda. To date, there is no other study that attempted to tackle this topic in the country. It was evident that even for the students who seem to be knowledgeable on AMR, proper use of antibiotics is not reflected in their daily life. However, the respondents showed an important interest to acquire more skills on antimicrobial use, prescribing and resistance. They were conscientious on the fact that, as future healthcare providers, they would participate in the fight against AMR starting from their early education.

The findings of this study are in accordance with those reported in studies conducted in India respectively among medical, dental and pharmacy students; that suggested the review of curricula in undergraduate classes to boost their knowledge on antimicrobial use and prescribing [10, 14] . In fact, when current study participants were asked if “*Antibiotics are indicated to reduce any kind of pain and inflammation”,* a correct answer was obtained from only 41.9% of respondents. Additionally, 62.4% of the respondents strongly agreed that the antibiotics could be used to treat bacterial infections. A possible explanation for these results may be the lack of adequate resources to learn about antimicrobials. More so, differences may be attributed to the absence of strict and enforced laws and regulations on how antibiotics are prescribed and dispensed in retail pharmacies in Rwanda.

The present study revealed that only 59.8% of the students concur with the statement that antimicrobials are overused in Rwanda, a rate far lower than that (90%) reported in a study conducted in Democratic republic of Congo among medical students and medical doctors [9].

This discrepancy may simply be attributed to lack of information on antimicrobial use.

Concerning antimicrobial susceptibility testing, 88.2% of respondents stressed that it is important to perform the test before prescribing any antibiotic. These findings are in fact very encouraging given the fact that antimicrobials are overprescribed and that medical and dental students, as future prescribers they are aware that prescribing antibiotics in a non-controlled way is not a good practice. Instantly development of new educational strategies for healthcare students to fight AMR is needed at university level for changing antimicrobial prescription behaviors and ensuring proper antimicrobial usage.

Regarding attitudes and perceptions towards antimicrobial use and resistance, 15% of students reported to have used antibiotics to treat fever. These findings are in line with the study conducted in Trinidad and Tobag [25]. This can partially explain the high rate of students who reported to have used Aspirin as an antibiotic and the 27% of respondents who used antibiotics to treat cold and sore throat. These factors may explain the relatively good correlation between the use of antibiotics to treat common cold and flu and the failure to differentiate antibiotics from non-steroidal anti-inflammatory drugs (NSAIDs) and painkillers. Around 80% of respondents reported not to keep leftover antibiotics for use in the future or for treating colds and flu.

However, almost a half of all study participants reported that they can use antibiotics without a medical prescription, this is in agreement with previous findings which have proved that self-medication is not only done by non-medical students [26]. These findings are rather disappointing because self-medication is among the leading causes of AMR [8].

Despite the fact that Rwandans have access to a public insurance scheme with low copayments, the lengthy of the insurance clearings before getting a health service as well as the easy accessibility of community pharmacies compared to health facilities, may contribute to self- purchasing.

Results on attitudes and perceptions on antimicrobial use and resistance are also in agreement with previous studies which proved that healthcare students had knowledge on antimicrobials but demonstrated poor attitudes towards antimicrobial use and resistance [27]. This lack of correlation between knowledge and attitude towards antimicrobial resistance have been reported in several other studies including a Kosovo study which illustrated that pharmacy students have uncommendable behaviors with regards to self-medication despite of the good theoretical knowledge on AMR [28].

Students admitted that AMR is now a global threat and were aware of the burden that may result from its rise. This study is consistent with two studies conducted in Southern India and South Africa which reported that healthcare students are aware that inappropriate use of antimicrobials may be harmful to patients and can cause antimicrobial resistance [15, 27].

Almost all respondents reported that apart from learning about AMR from degree courses, they have heard about it from media. A survey conducted at the University of Torino revealed that healthcare students do not have a course specifically focusing on antibiotic resistance [17]. This matches with the reality observed in healthcare students’ curricula in Rwanda, the topic of AMR is discussed in several degree courses, such as “Pharmacology” or “Infectious diseases course” in which likely the antibiotics can be discussed; but there is no specific course focusing on antimicrobial resistance except for Pharmacy students which may explain their better knowledge on AMR compared to other healthcare students.

Moreover, strengthening antimicrobial stewardships in all health facilities would create an continual platform of discussion for fresh prescribers and pharmacists to draw evidence-based solutions on antimicrobial resistance [29, 30].

## Conclusion

This study showed a good knowledge of healthcare students regarding AMR but this was not reflected into their attitudes towards antimicrobial resistance. They fail to apply their knowledge in the real life. Students in health career tend to practice self-medication pretending to know which medication to take even without consulting a healthcare provider. The present study included medical, dental and pharmacy schools only, the future studies can consider other healthcare students.

The present study recommends educational interventions to enhance the knowledge and understanding of Antimicrobial resistance and prescription including reviewing the curricula and introducing the course of Antimicrobial resistance in healthcare field curricula starting in lower levels. Finally, strict policies should be put in place to regulate the flow of antibiotics and to prevent the purchase of antibiotics without a medical prescription.

## Data Availability

The datasets analyzed during the current study are available from the corresponding author on reasonable request.

## Abbreviations

AMR: Antimicrobial Resistance
FIP: International pharmaceutical Federation
LMIC: Low middle income countries
NSAIDs: Nonsteroidal anti-inflammatory drugs
SPSS: Statistical Package for the Social Sciences
WHO: World Health Organization
